# Supplementary figures and images for: Ptbp1 is not required for retinal neurogenesis and cell fate specification
Source: eLife. 2025 Dec 4;14:RP108331. doi: 10.7554/eLife.108331 (PMC12677894; doi:10.7554/eLife.108331)

## Supplementary File 2

### A. Rod Specific Exons

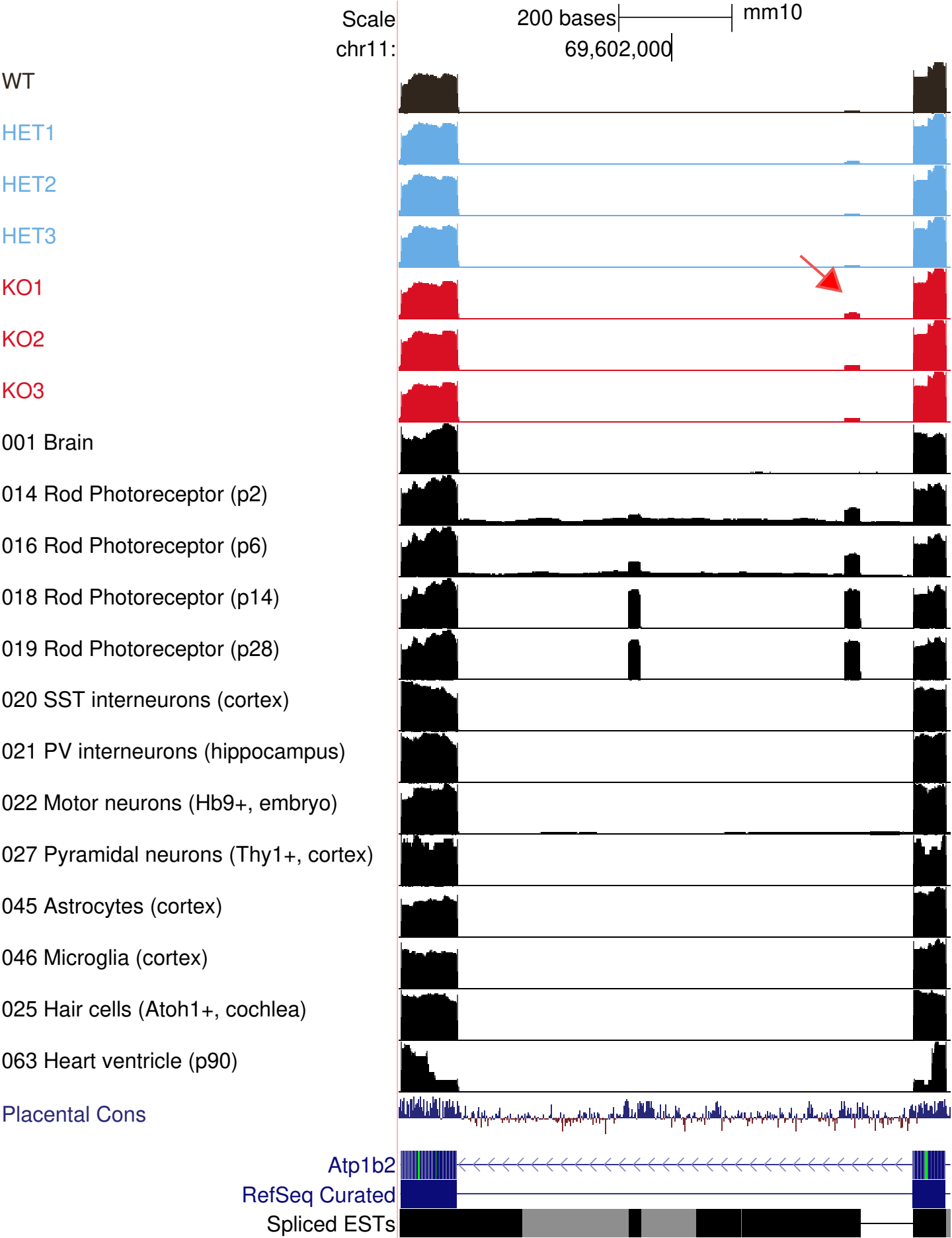

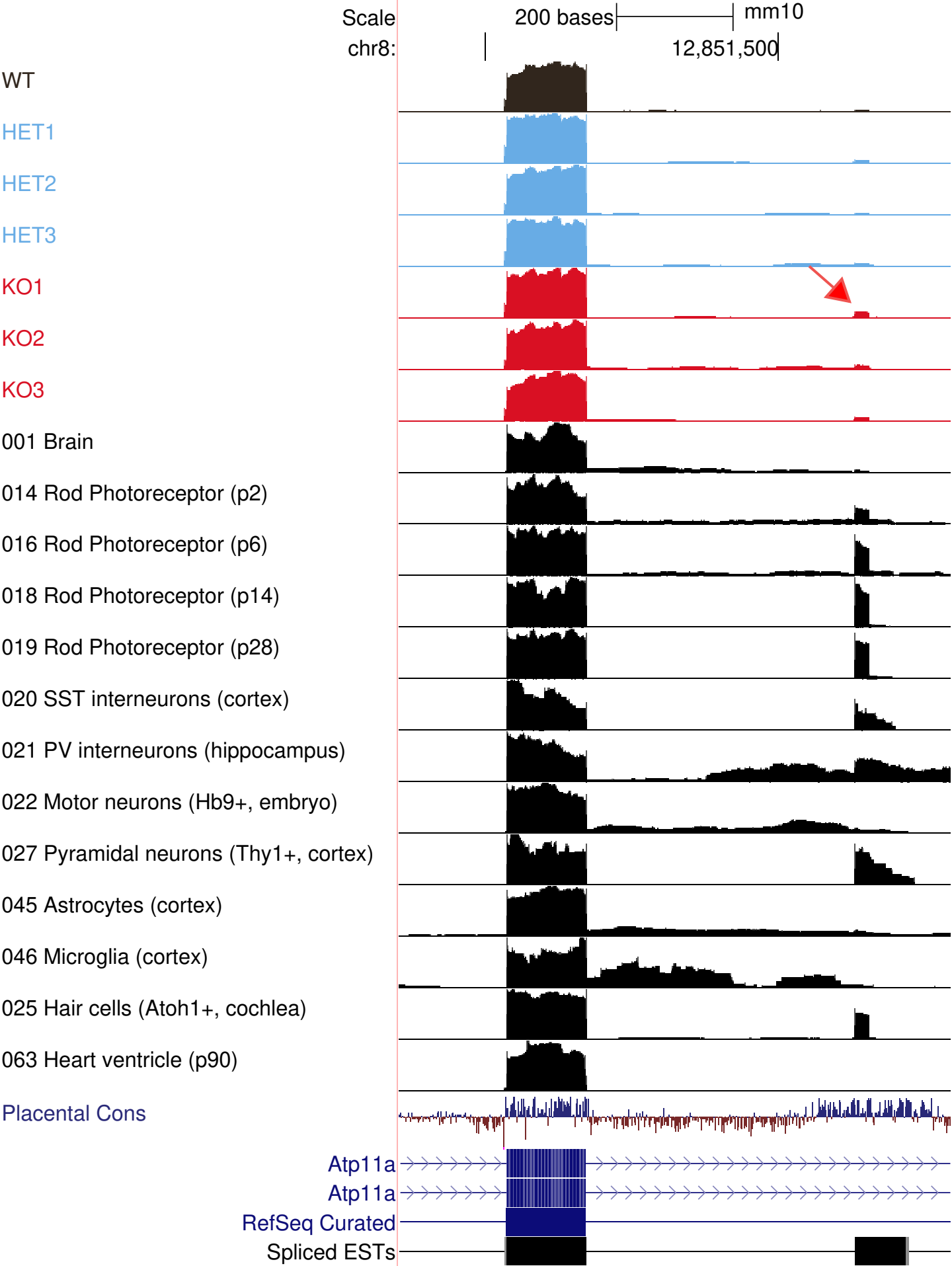

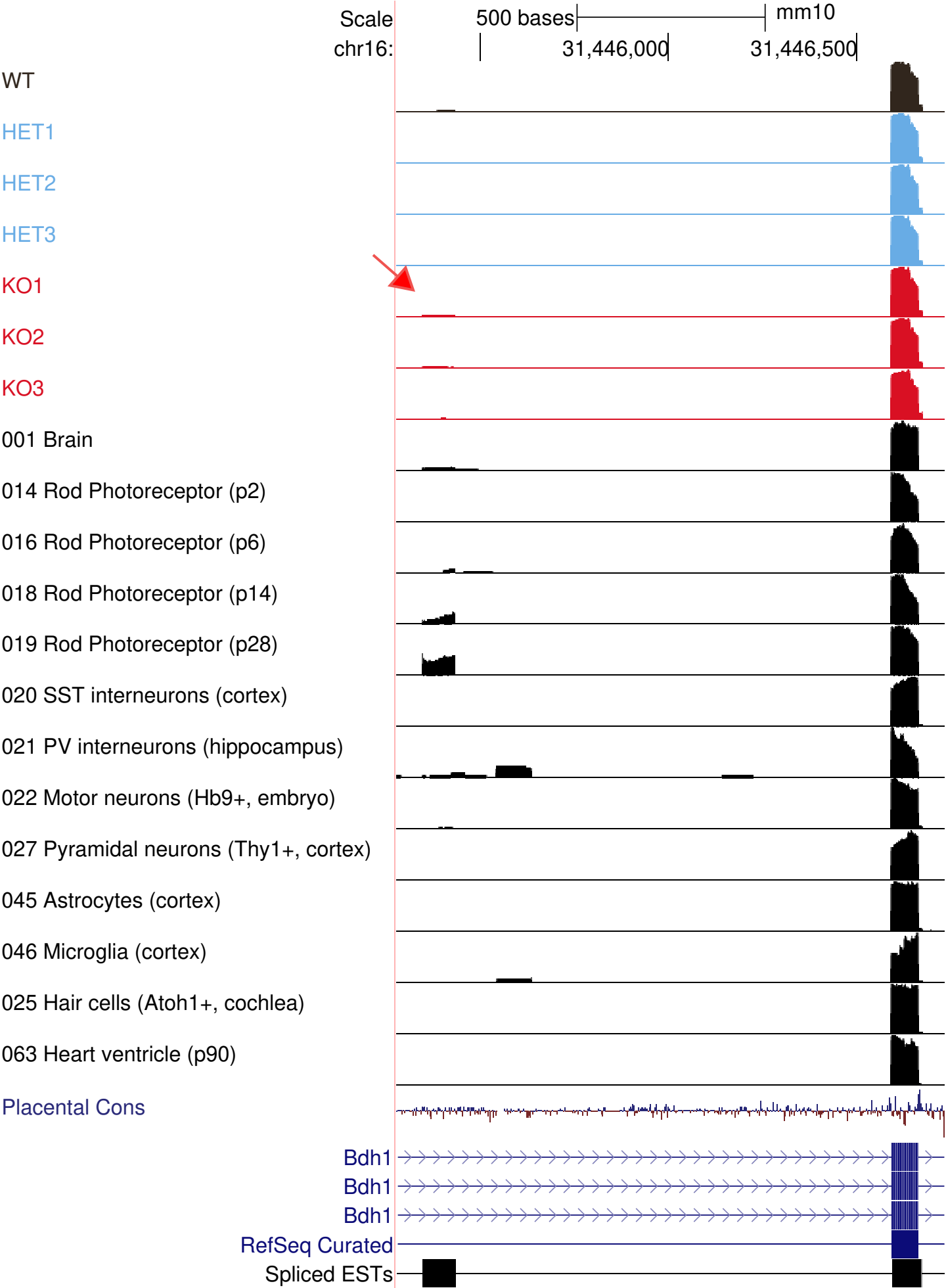

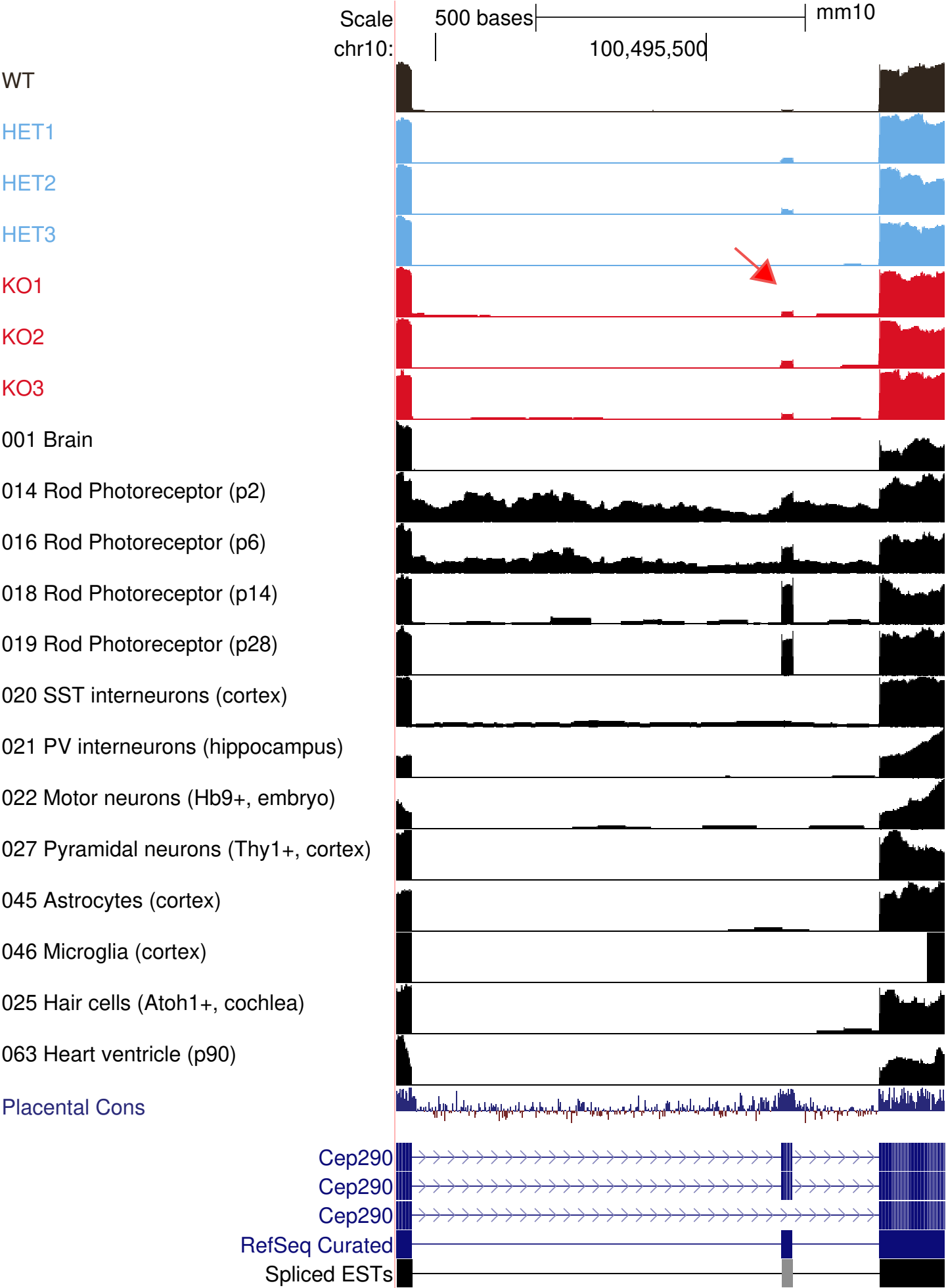

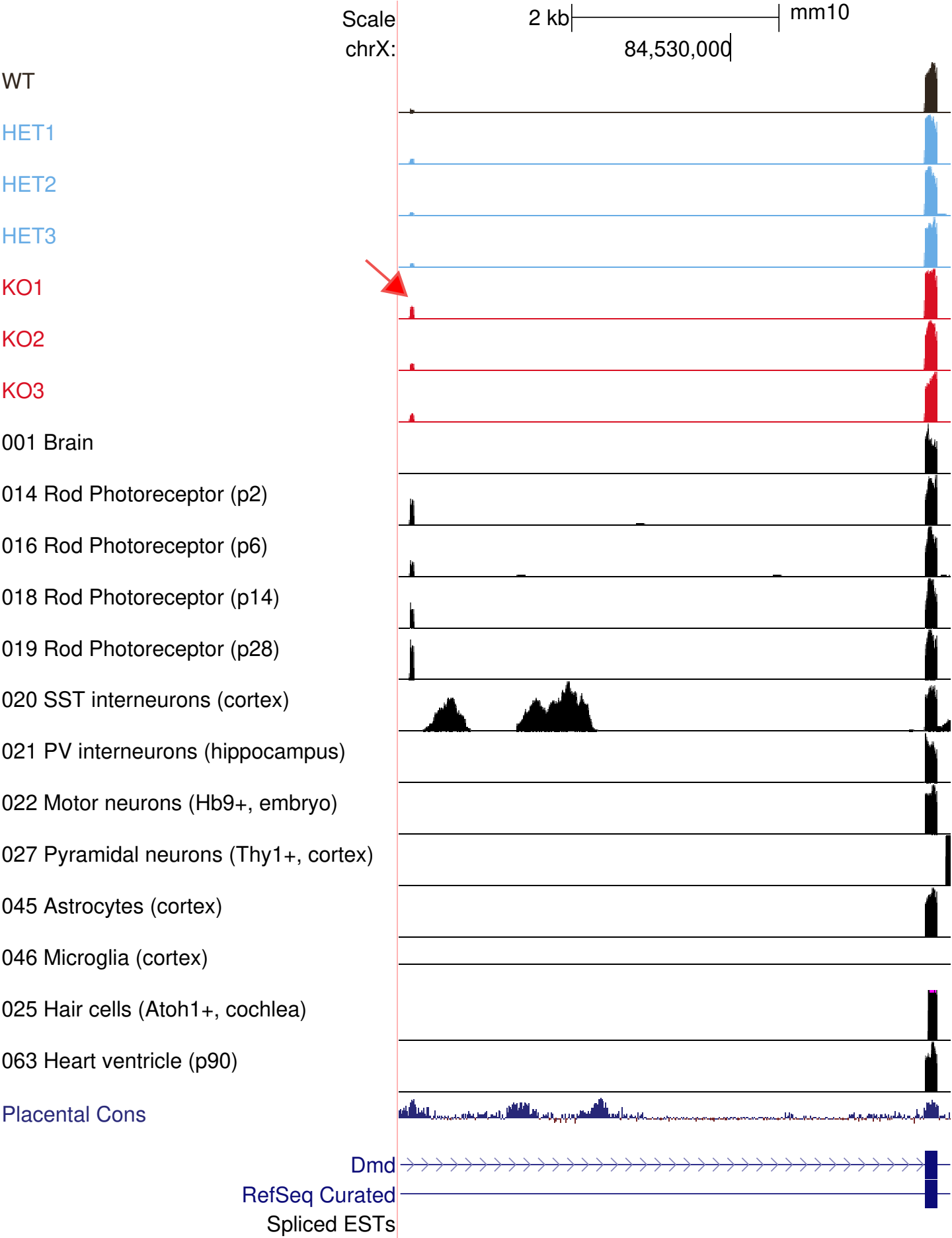

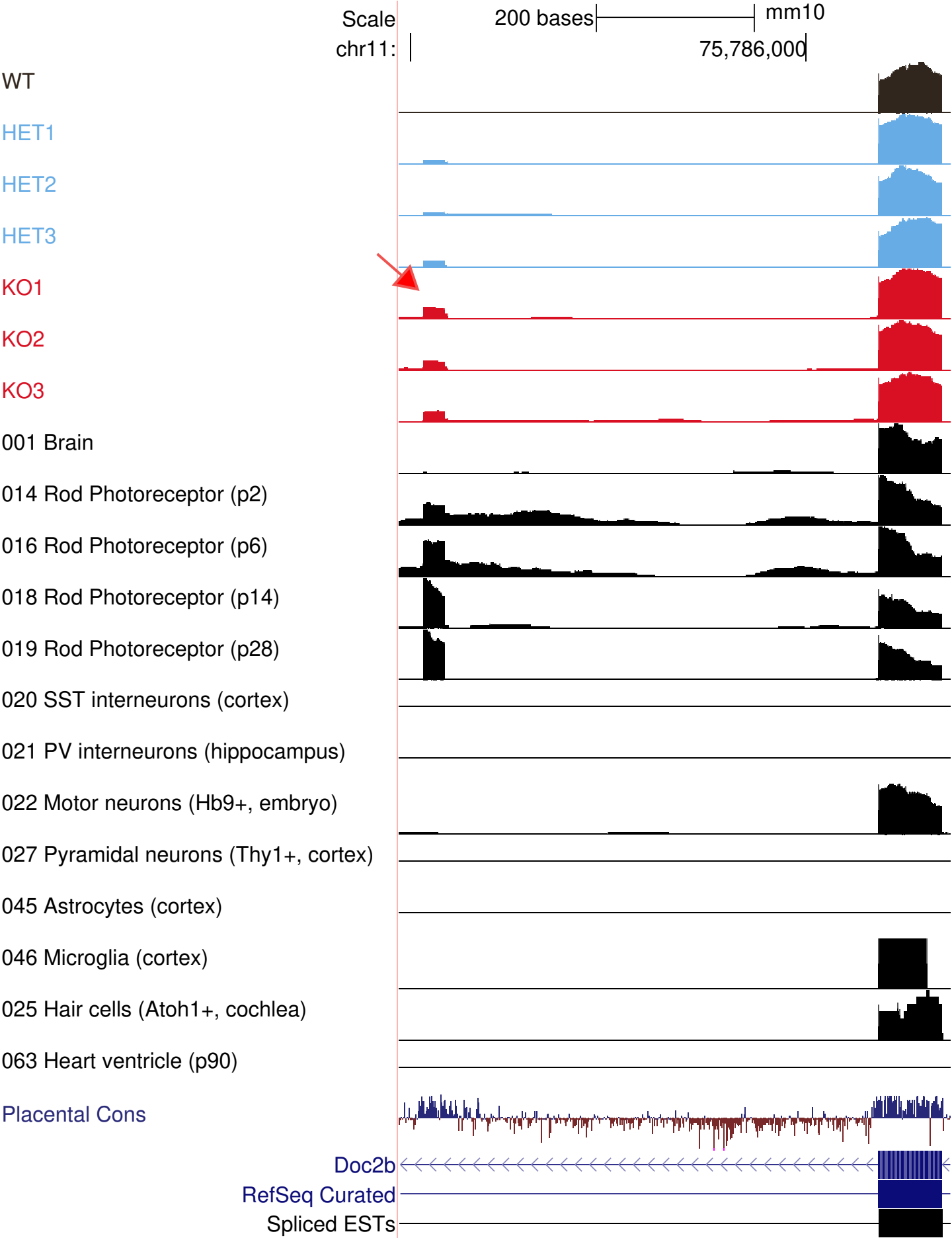

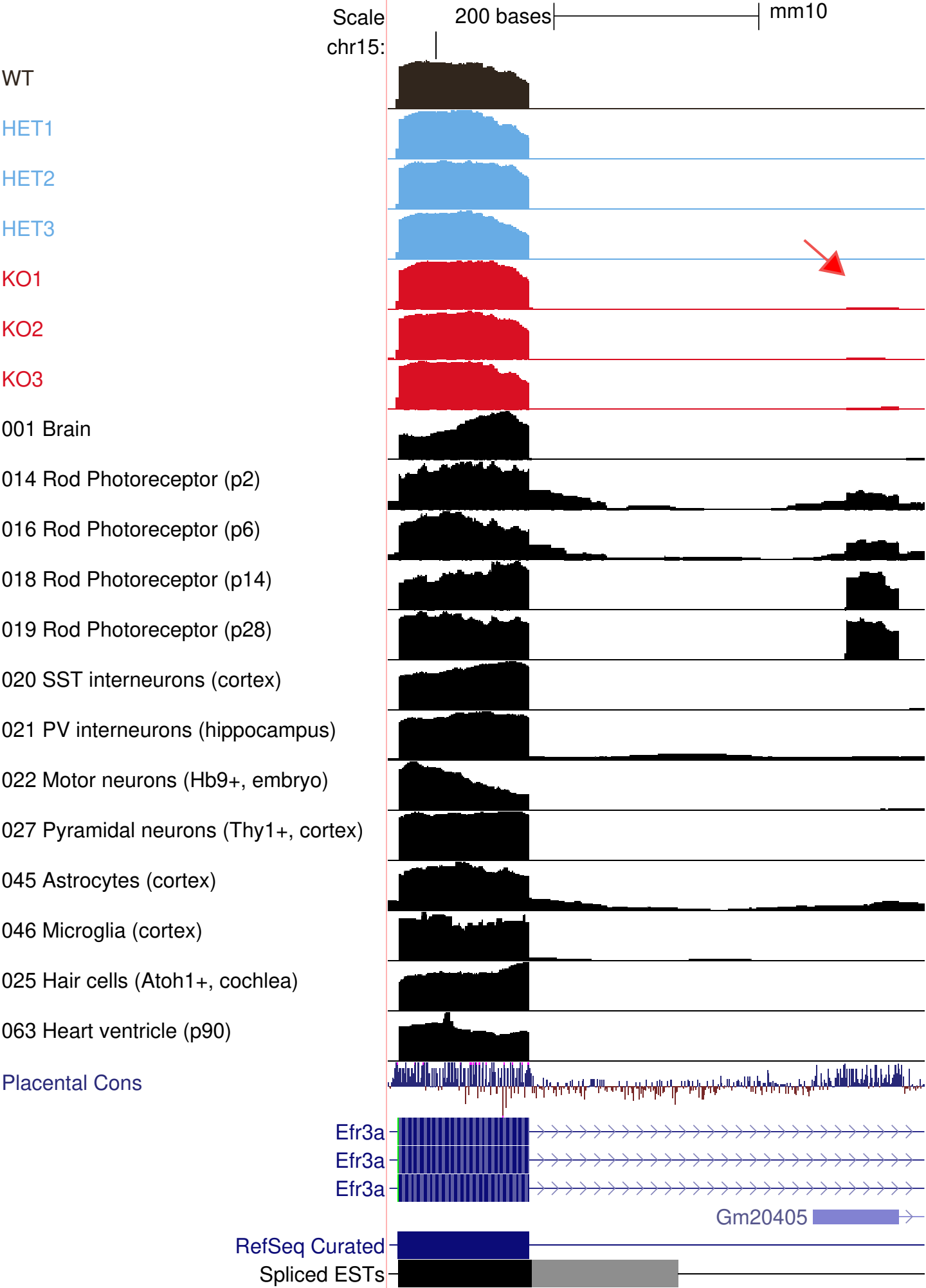

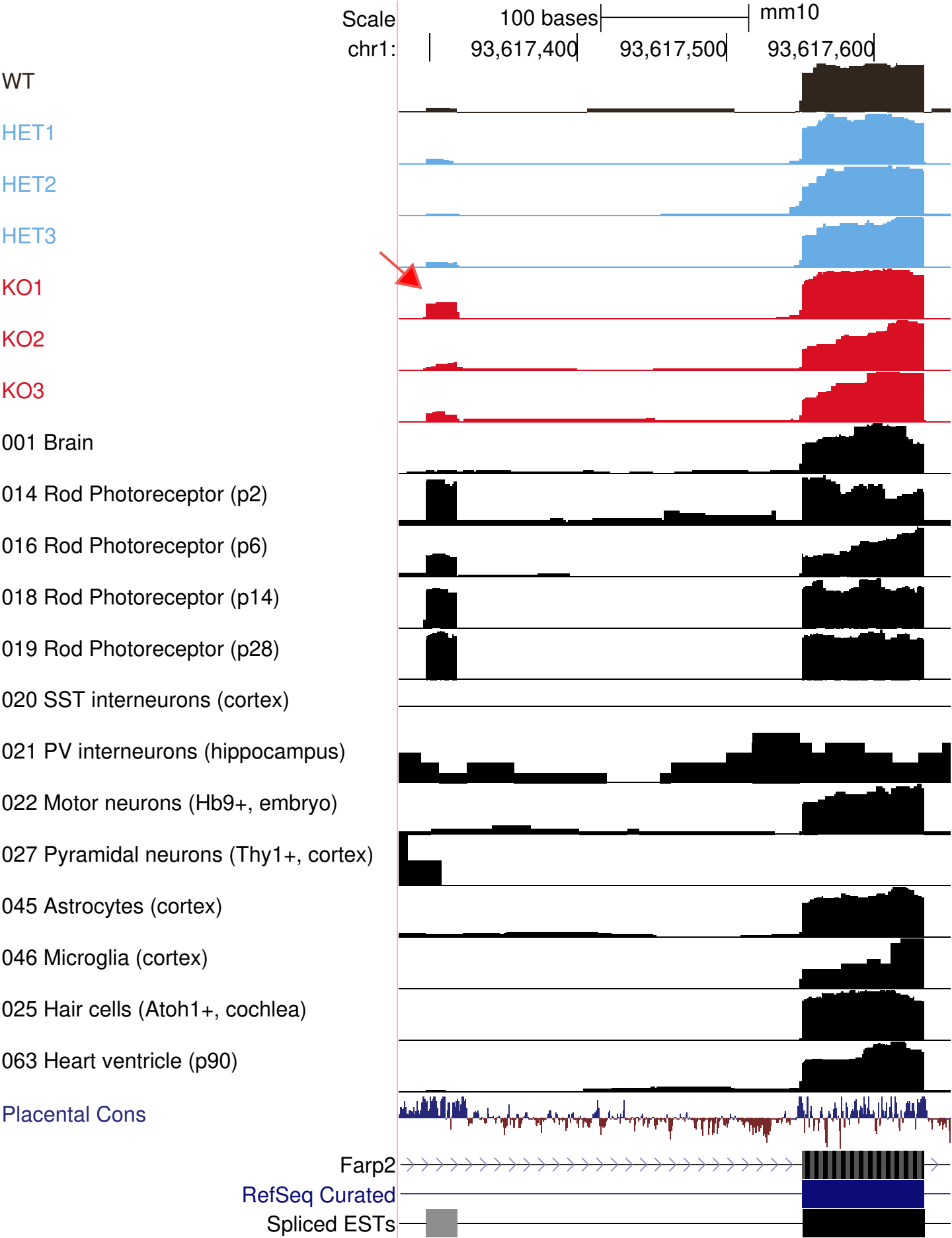

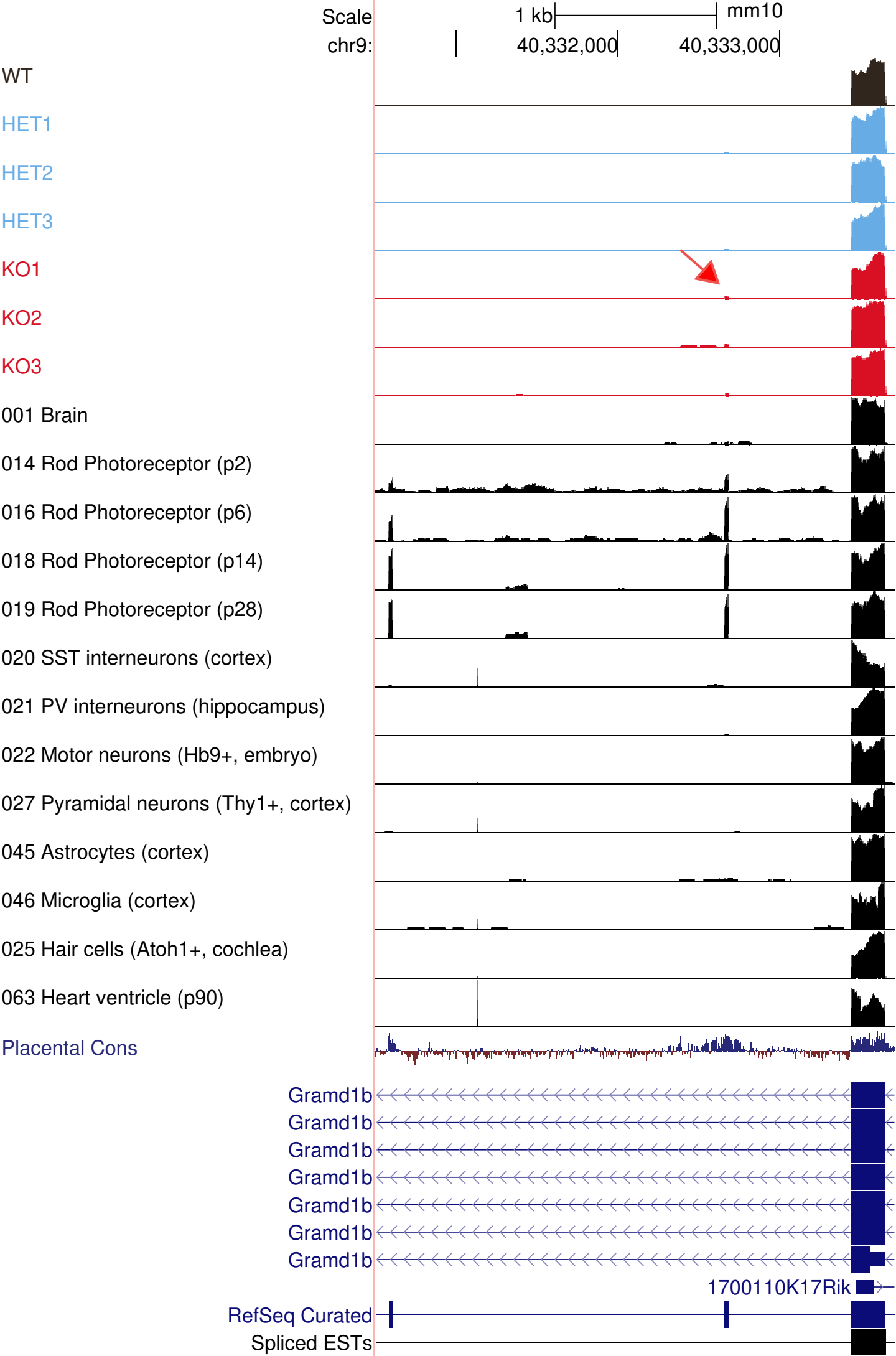

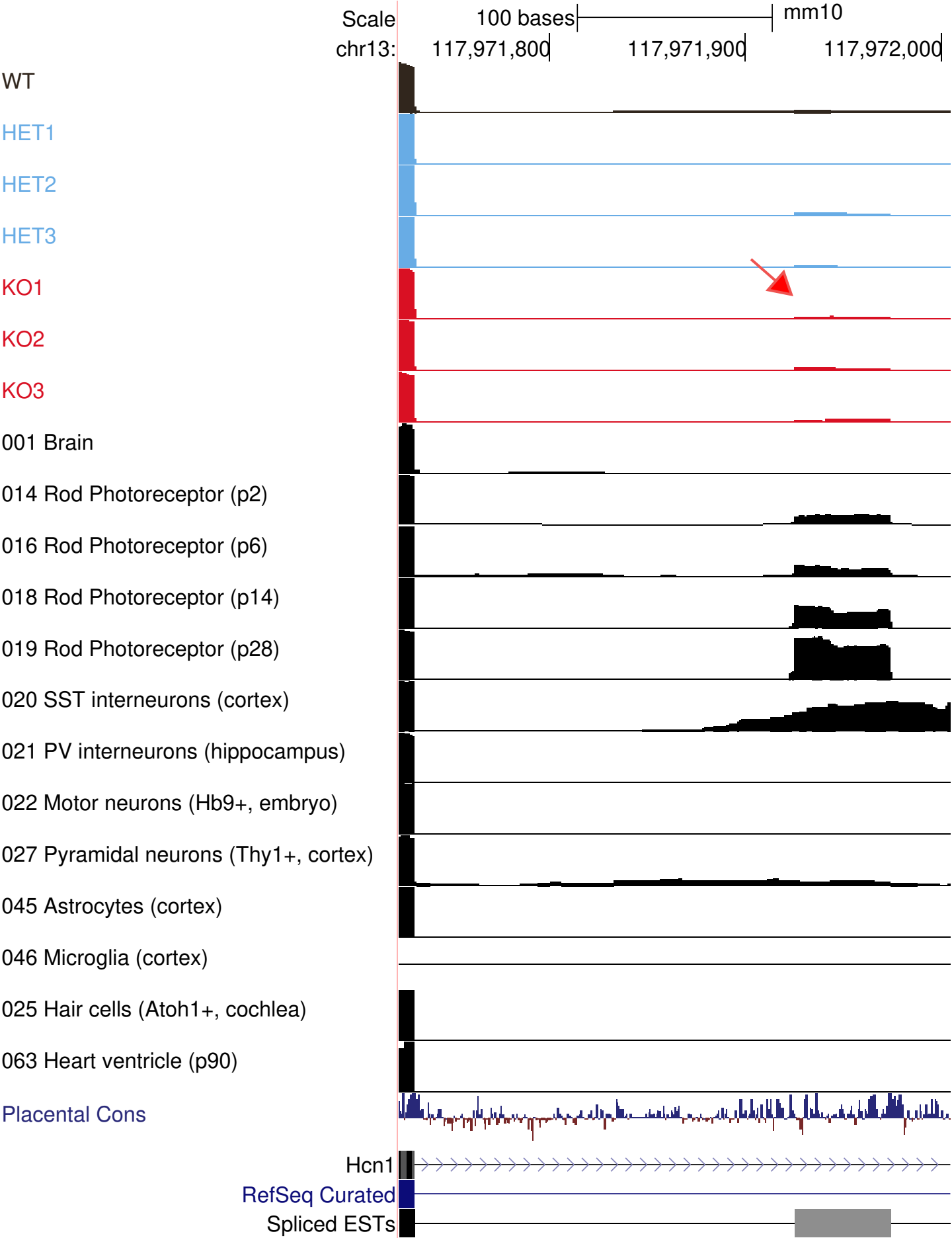

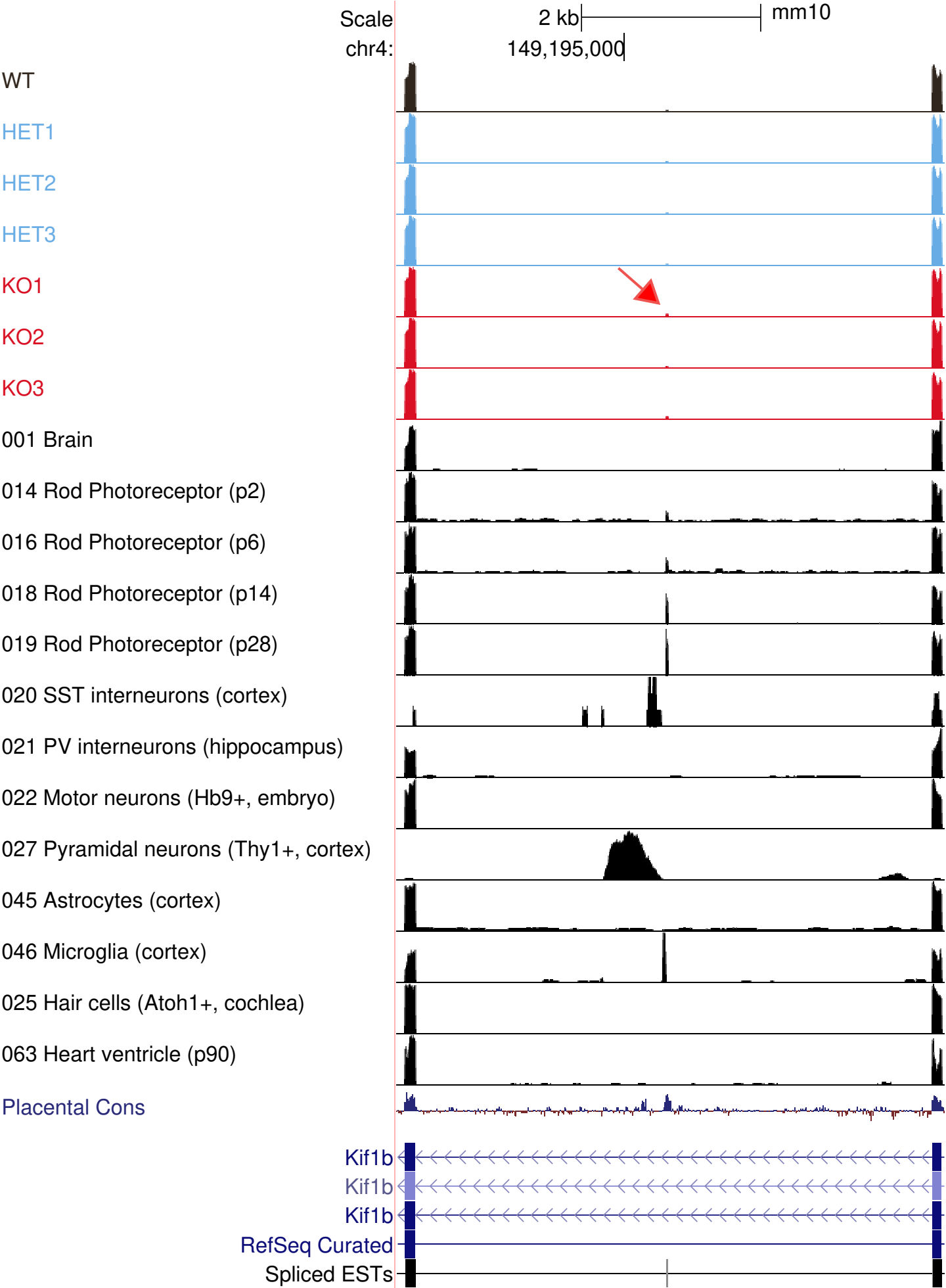

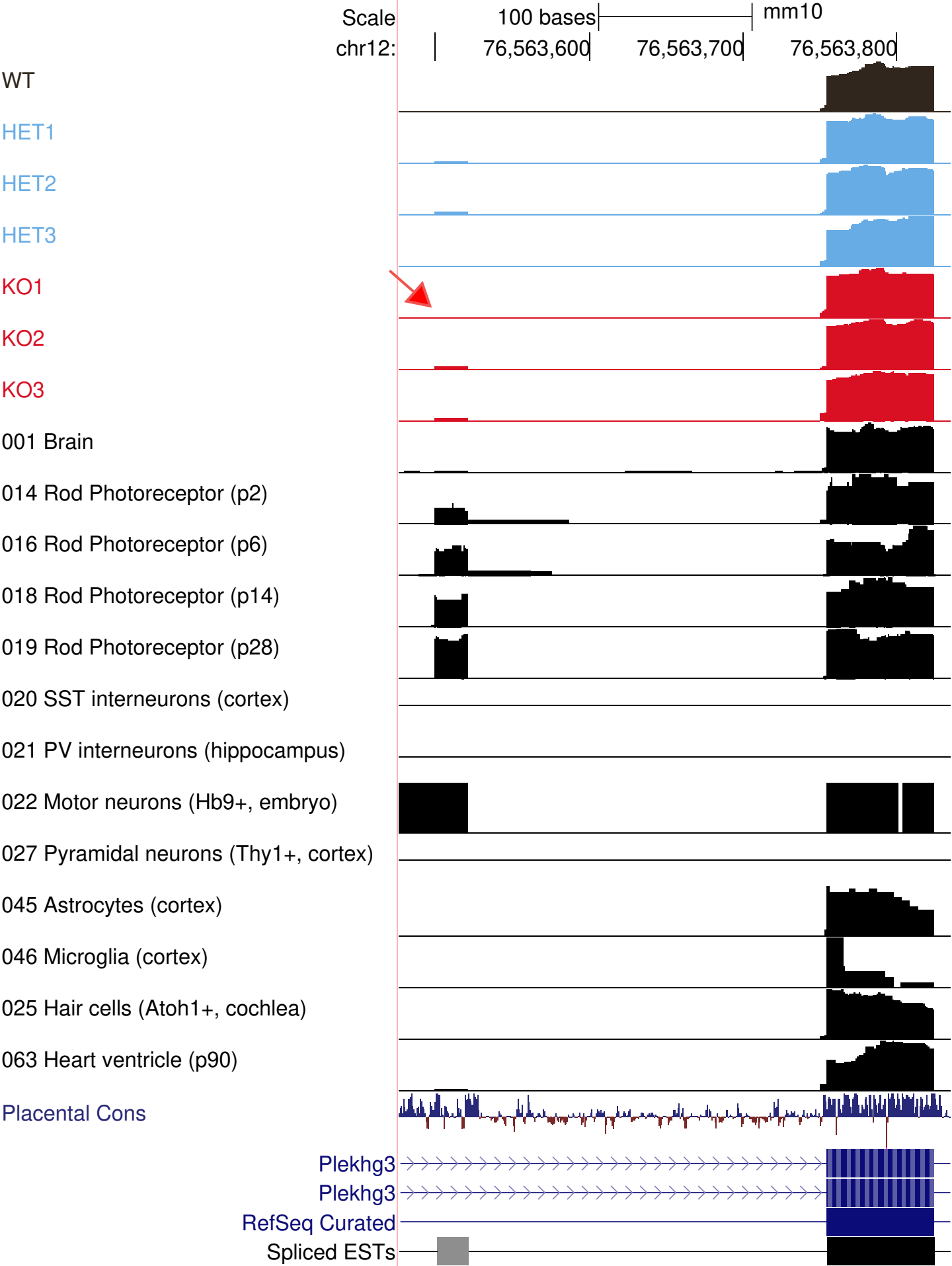

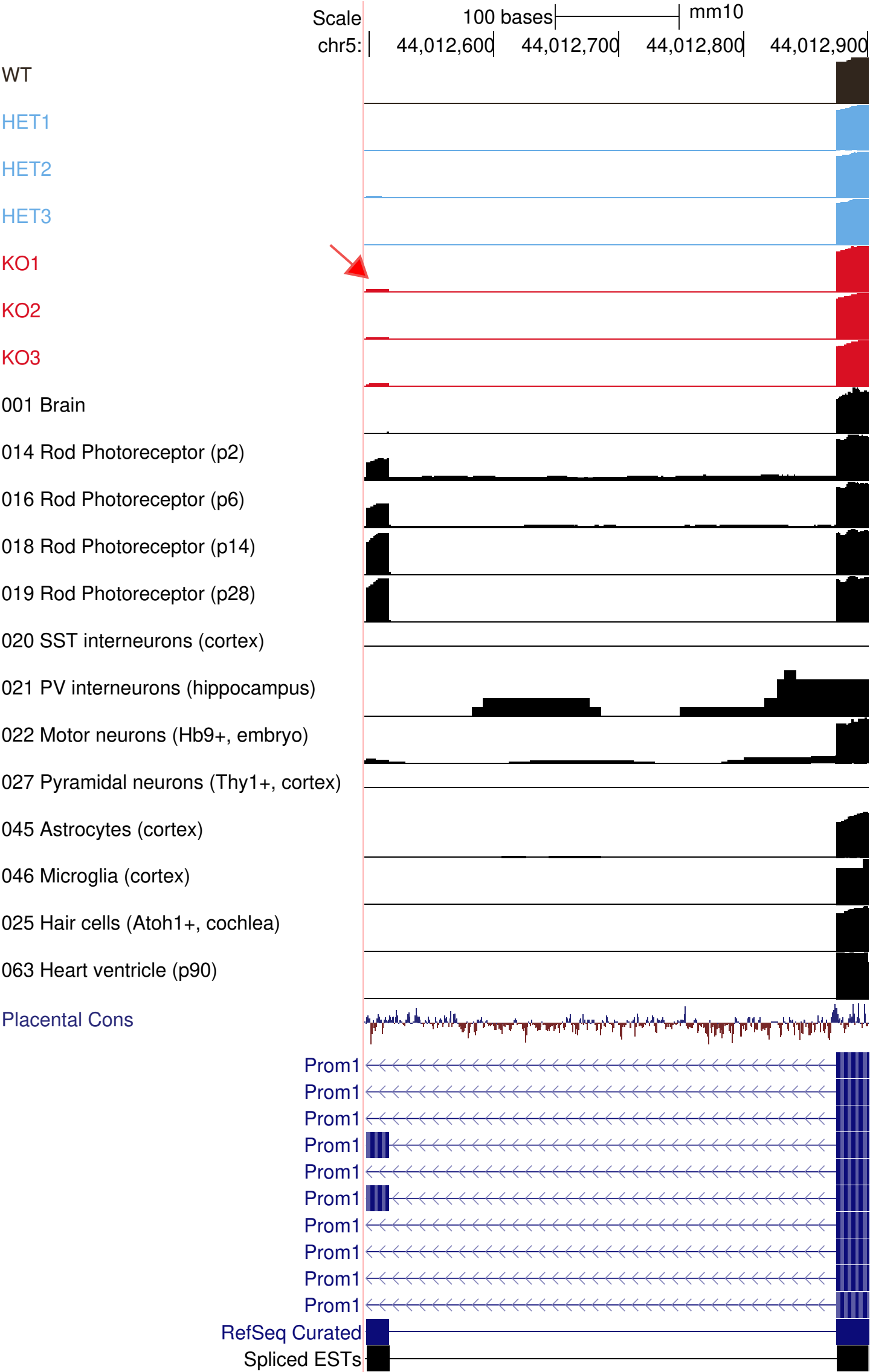

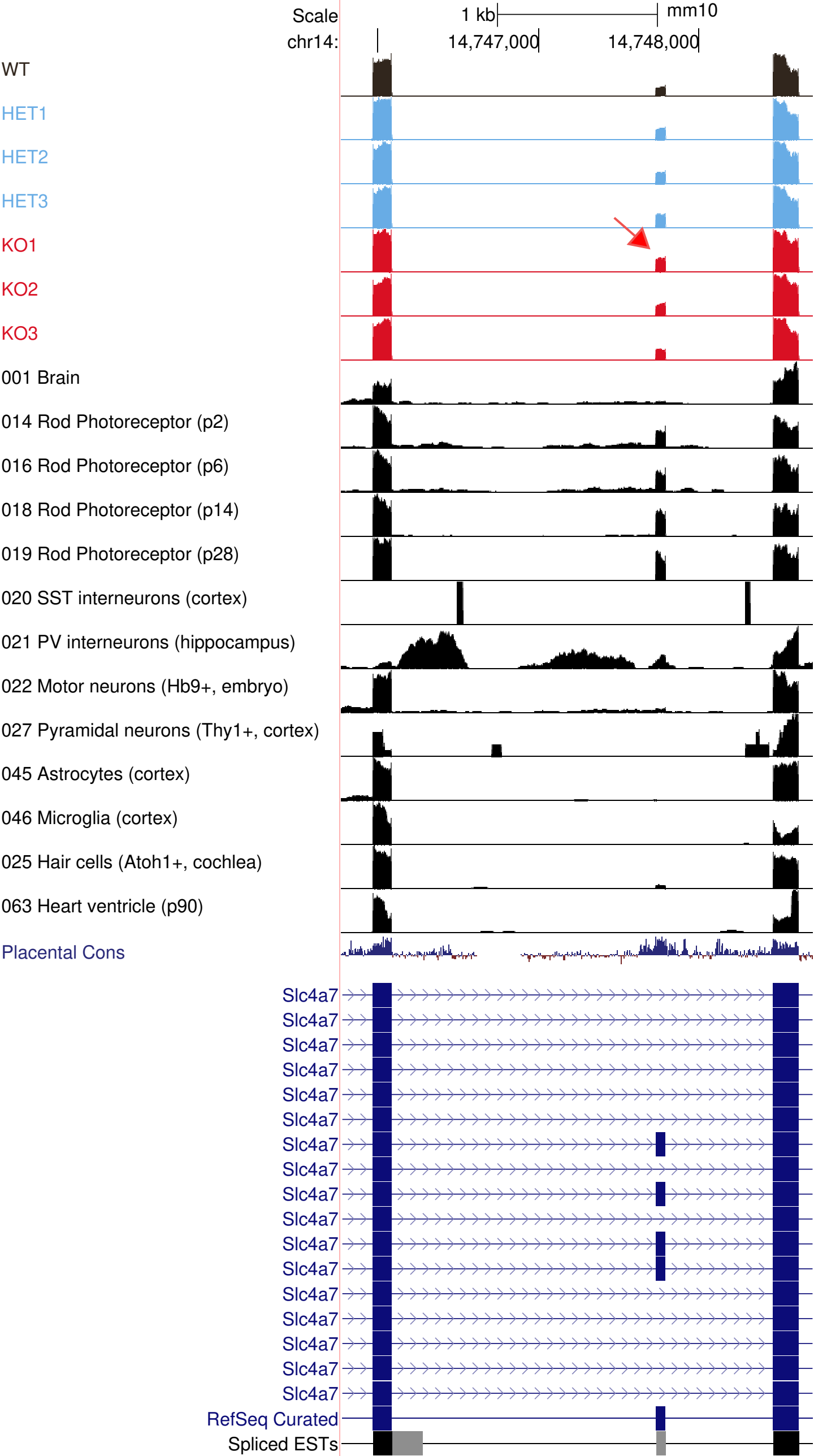

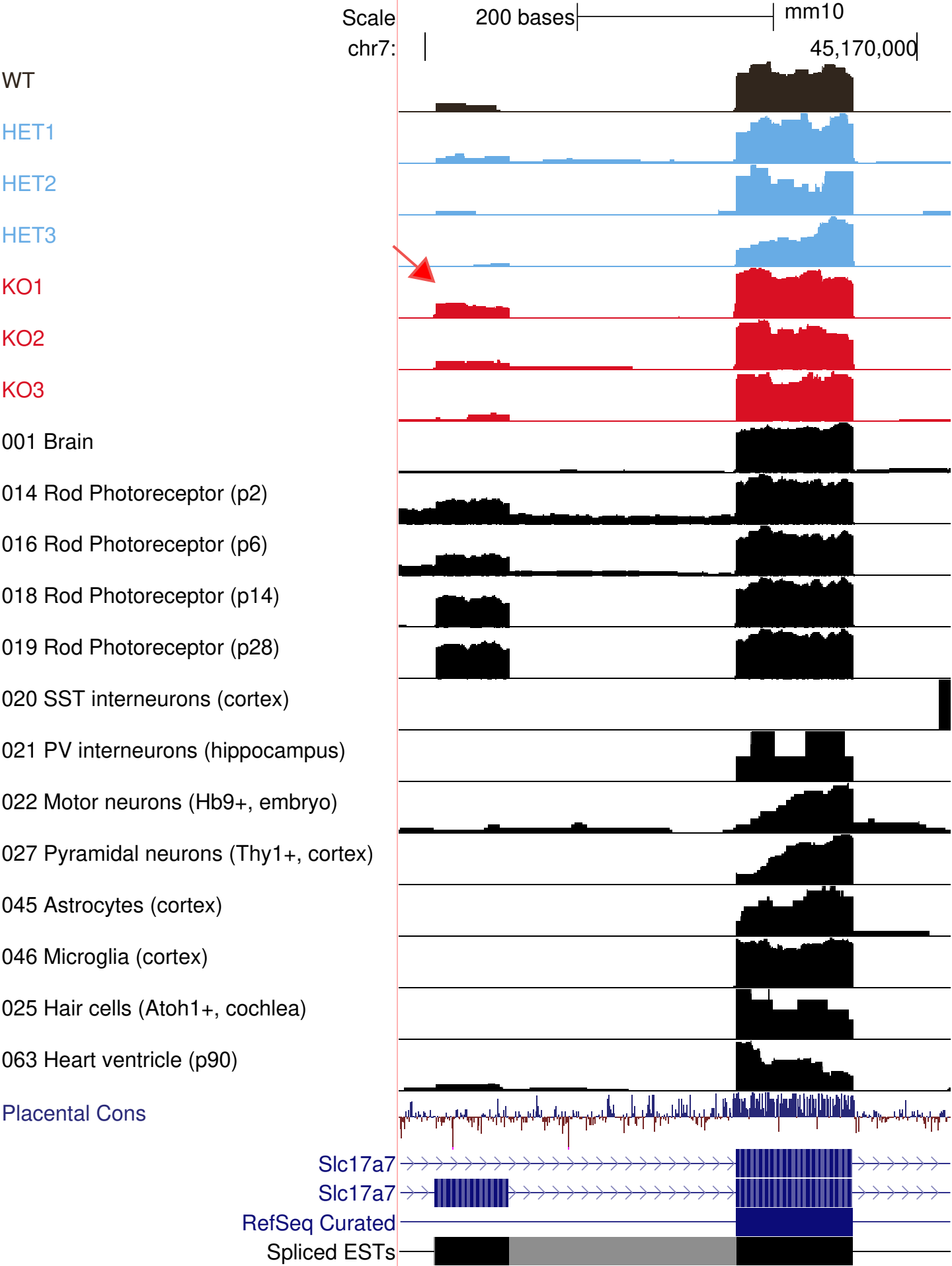

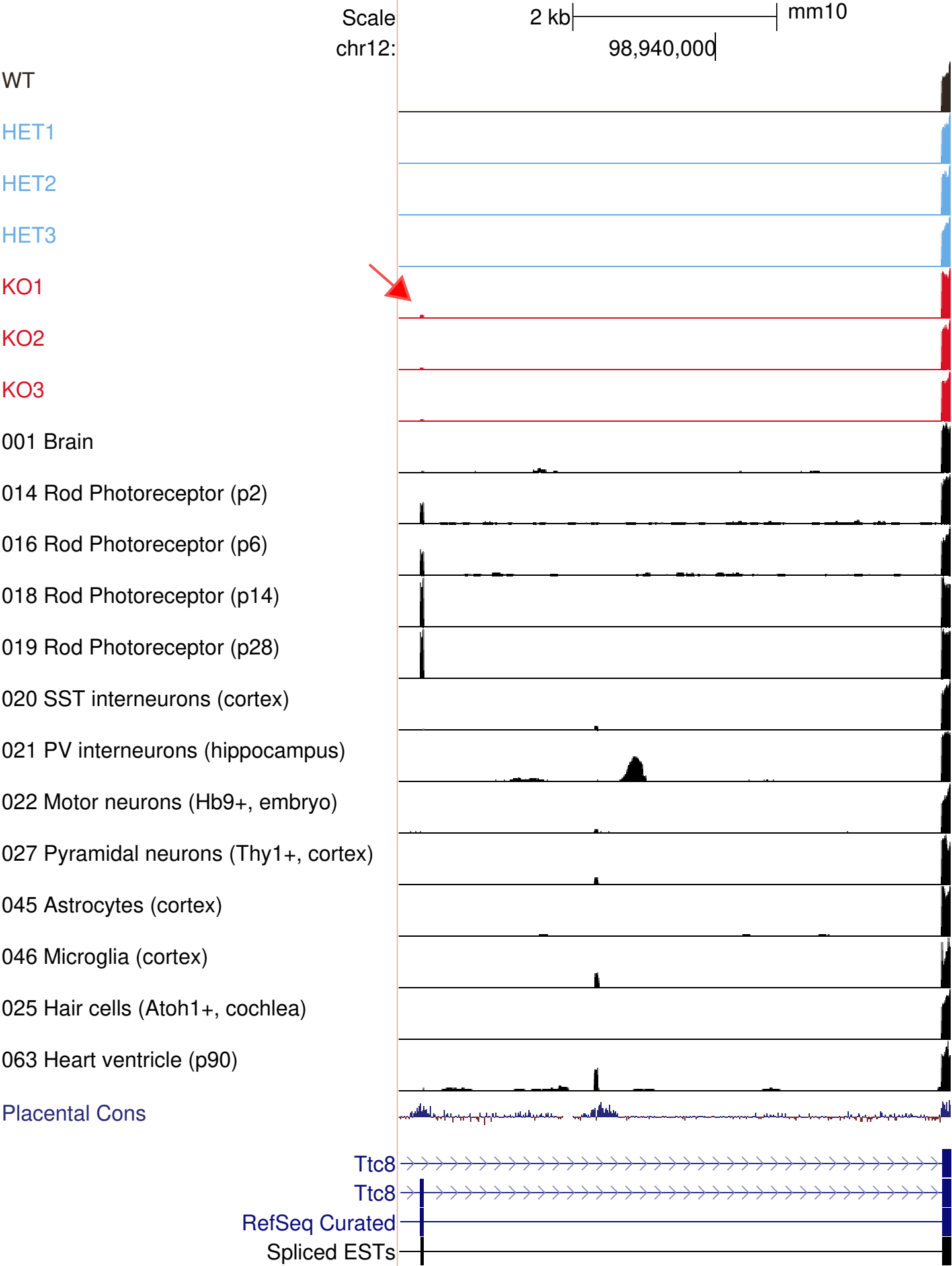

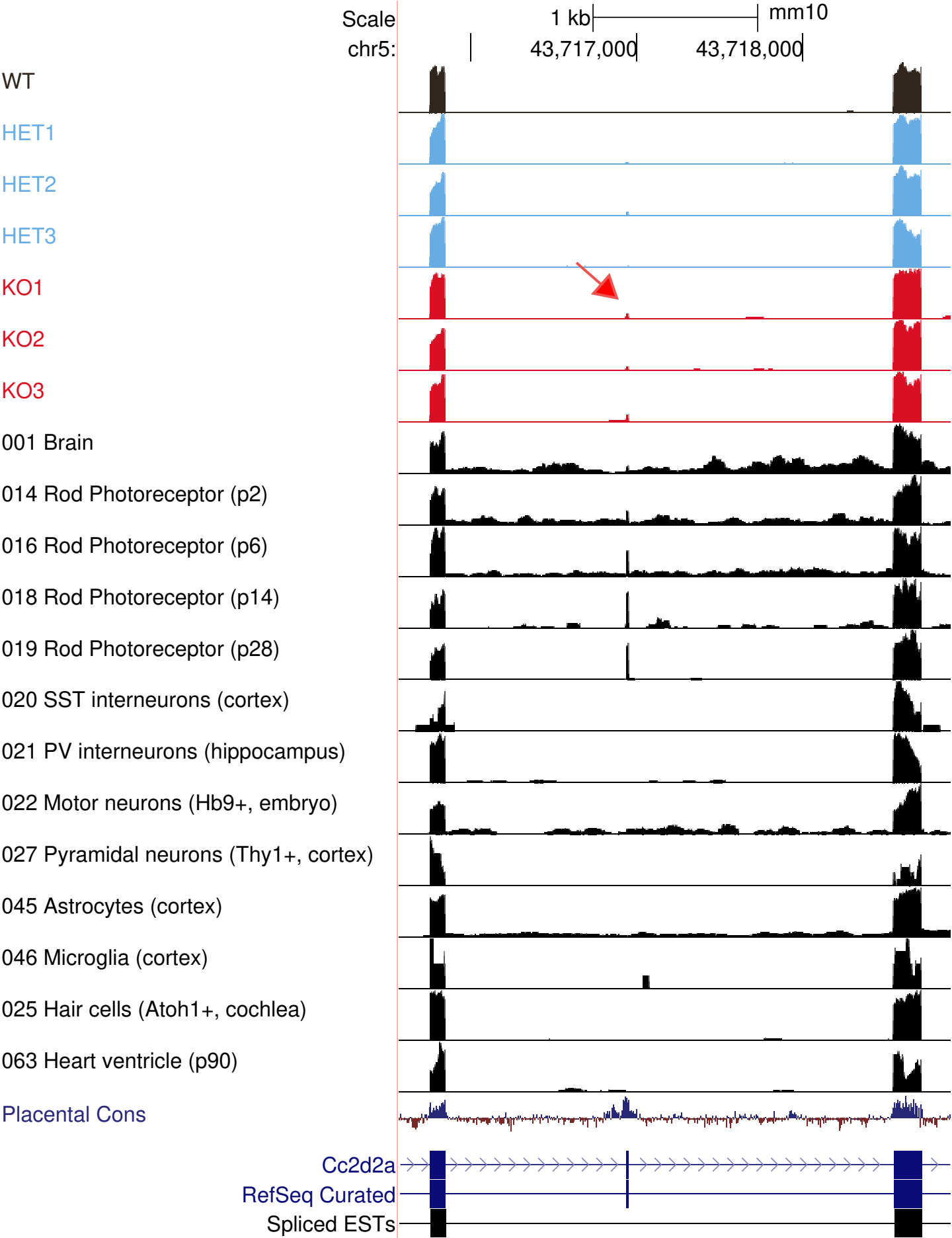

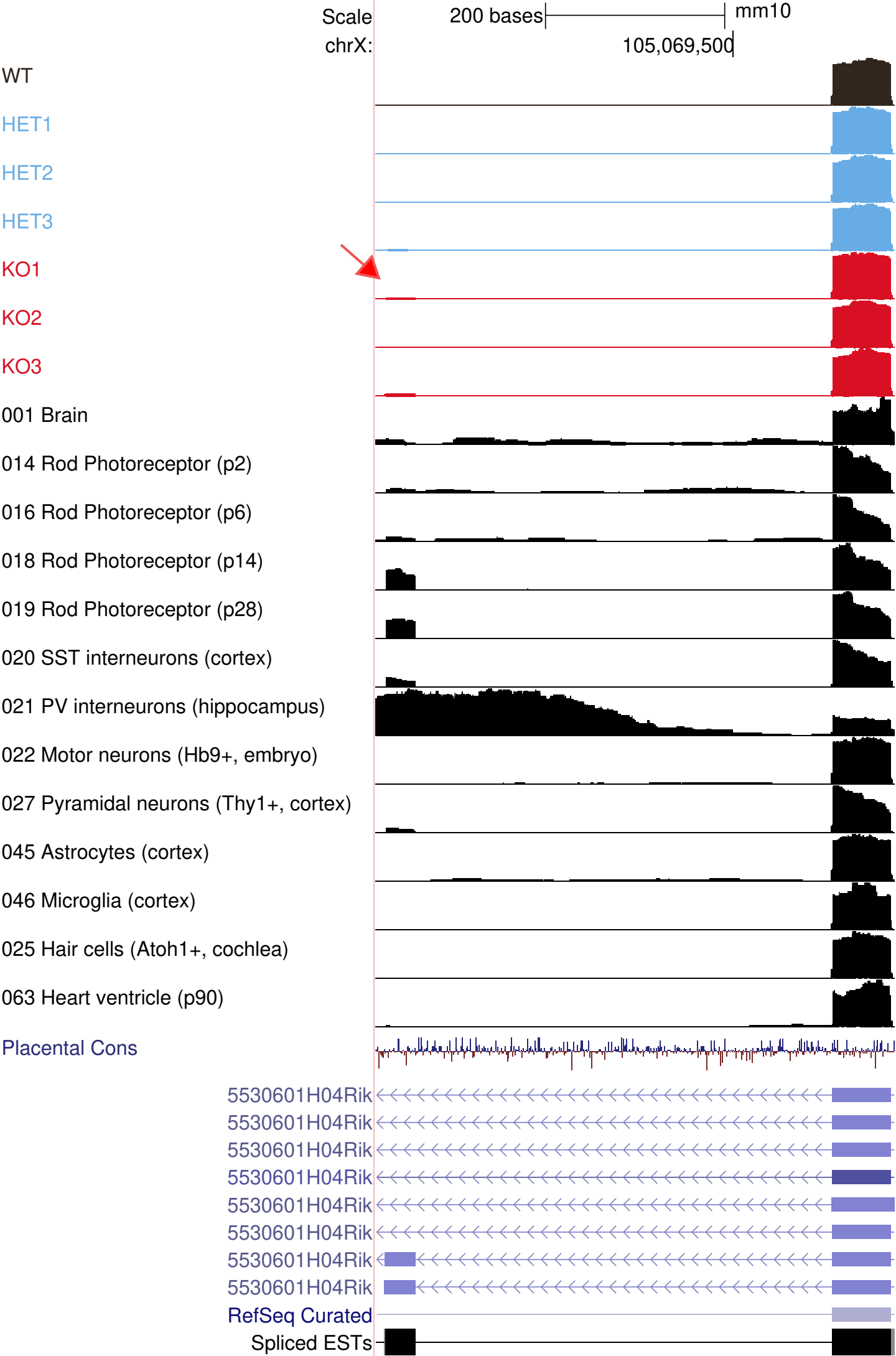

## Extended Dataset 3

### B. Neuron Specific Exons

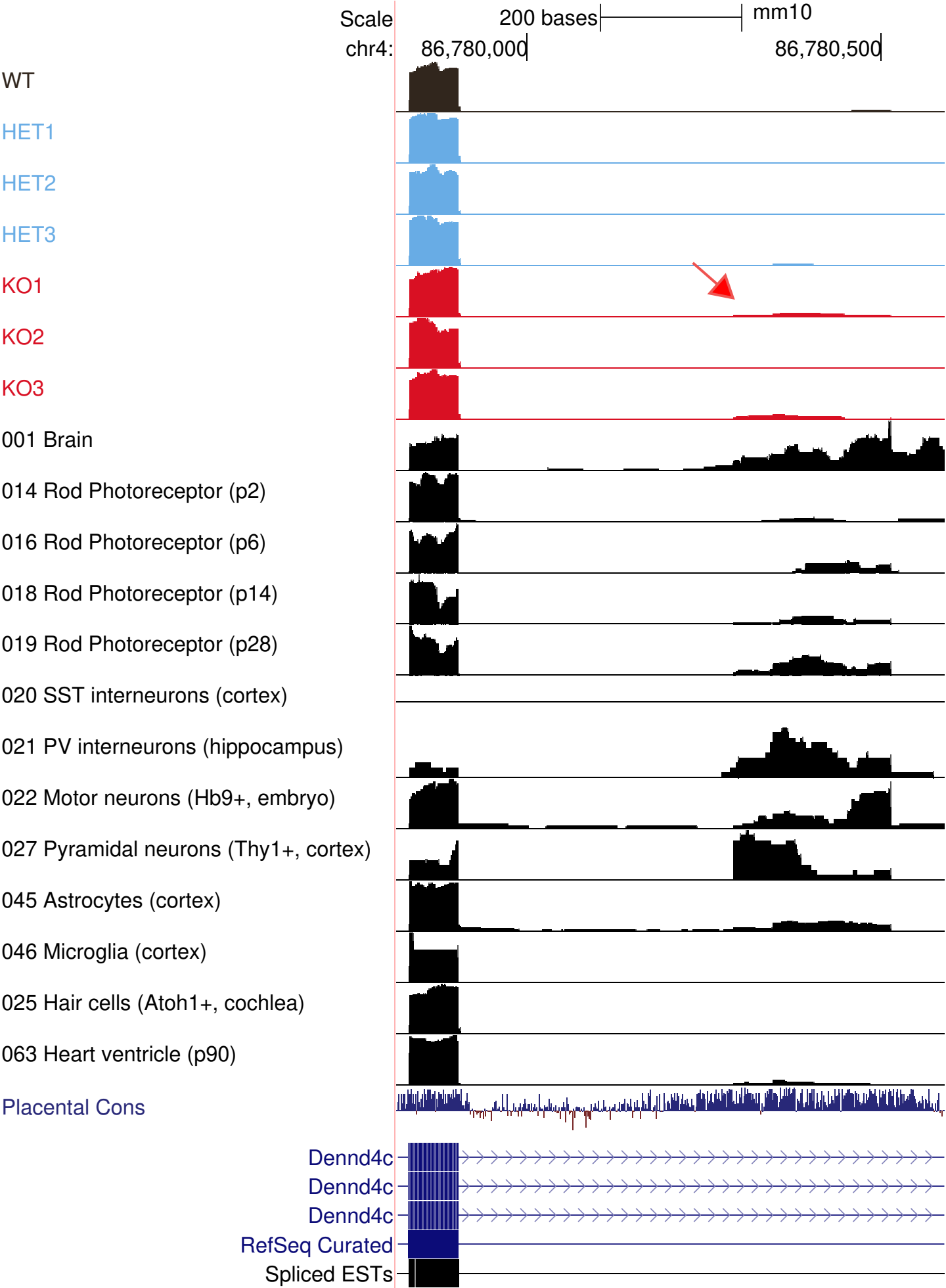

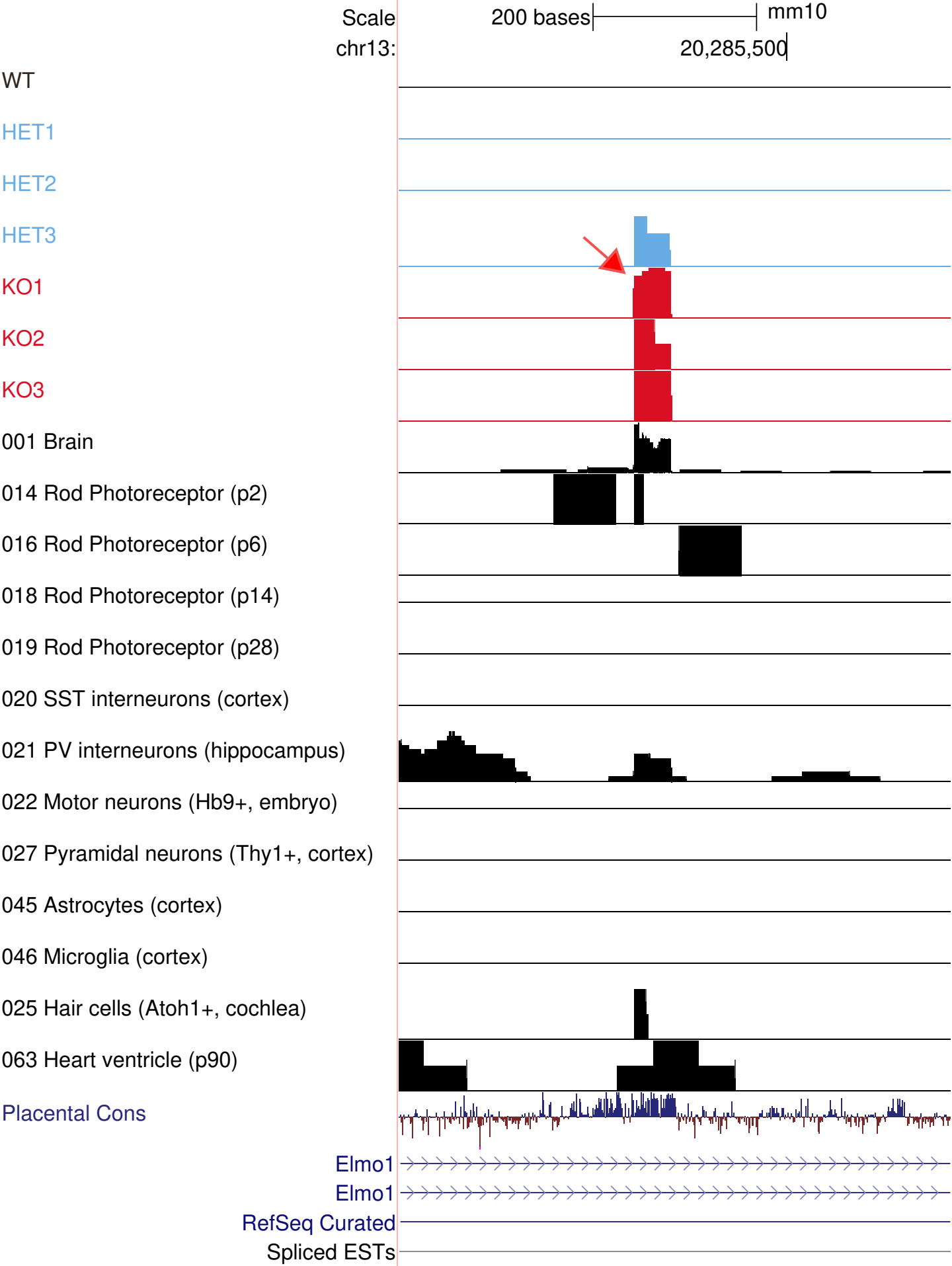

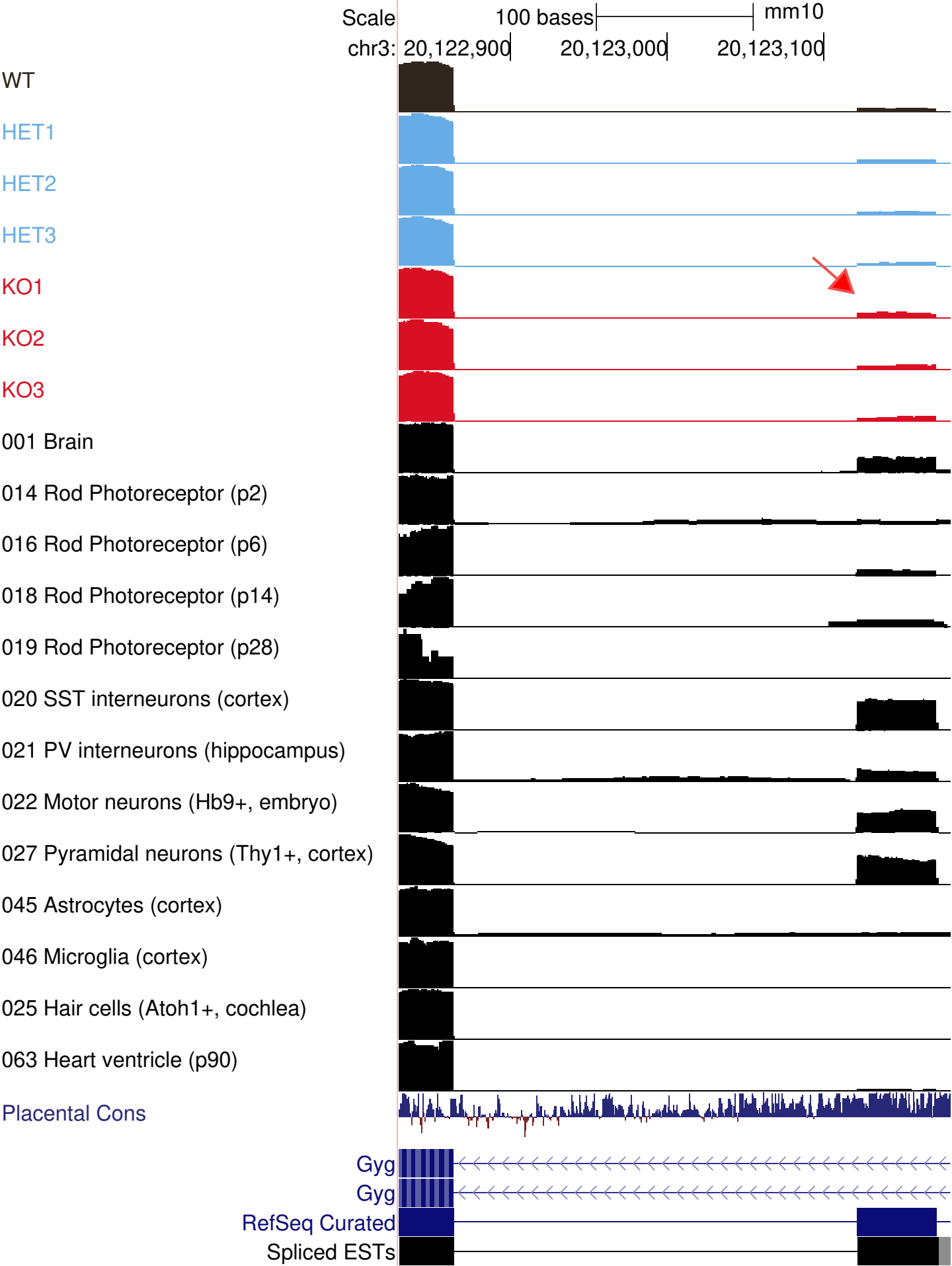

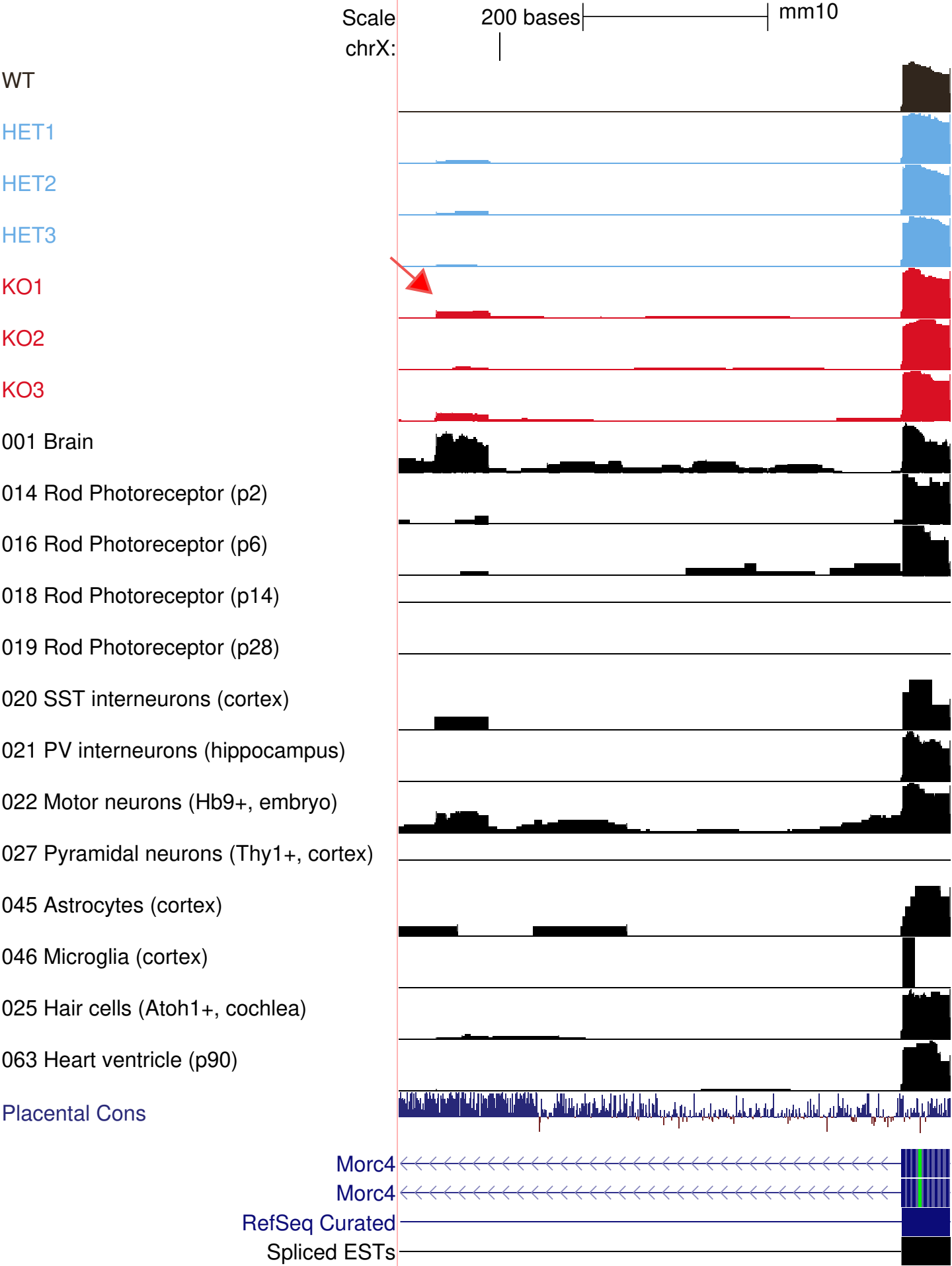

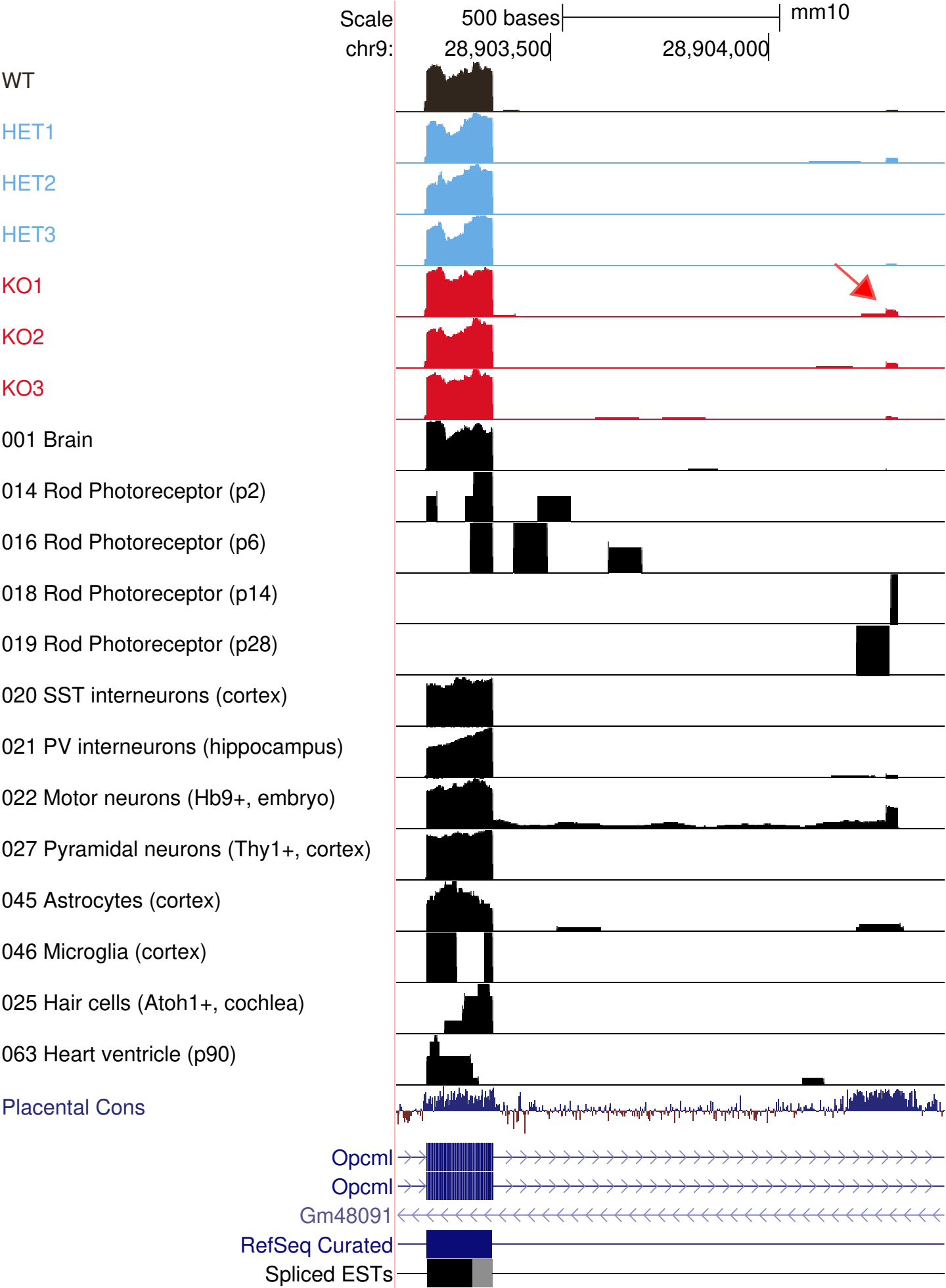

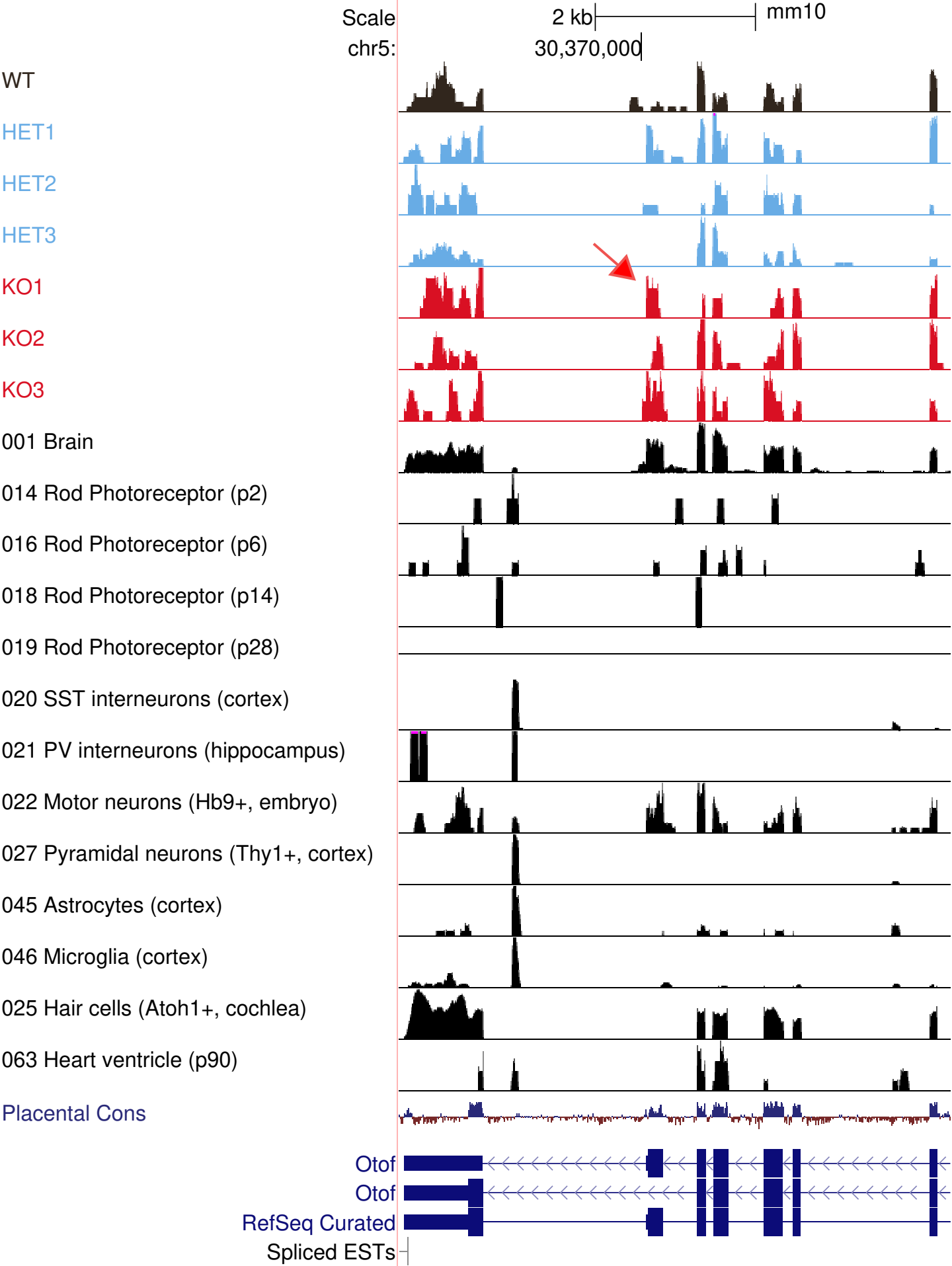

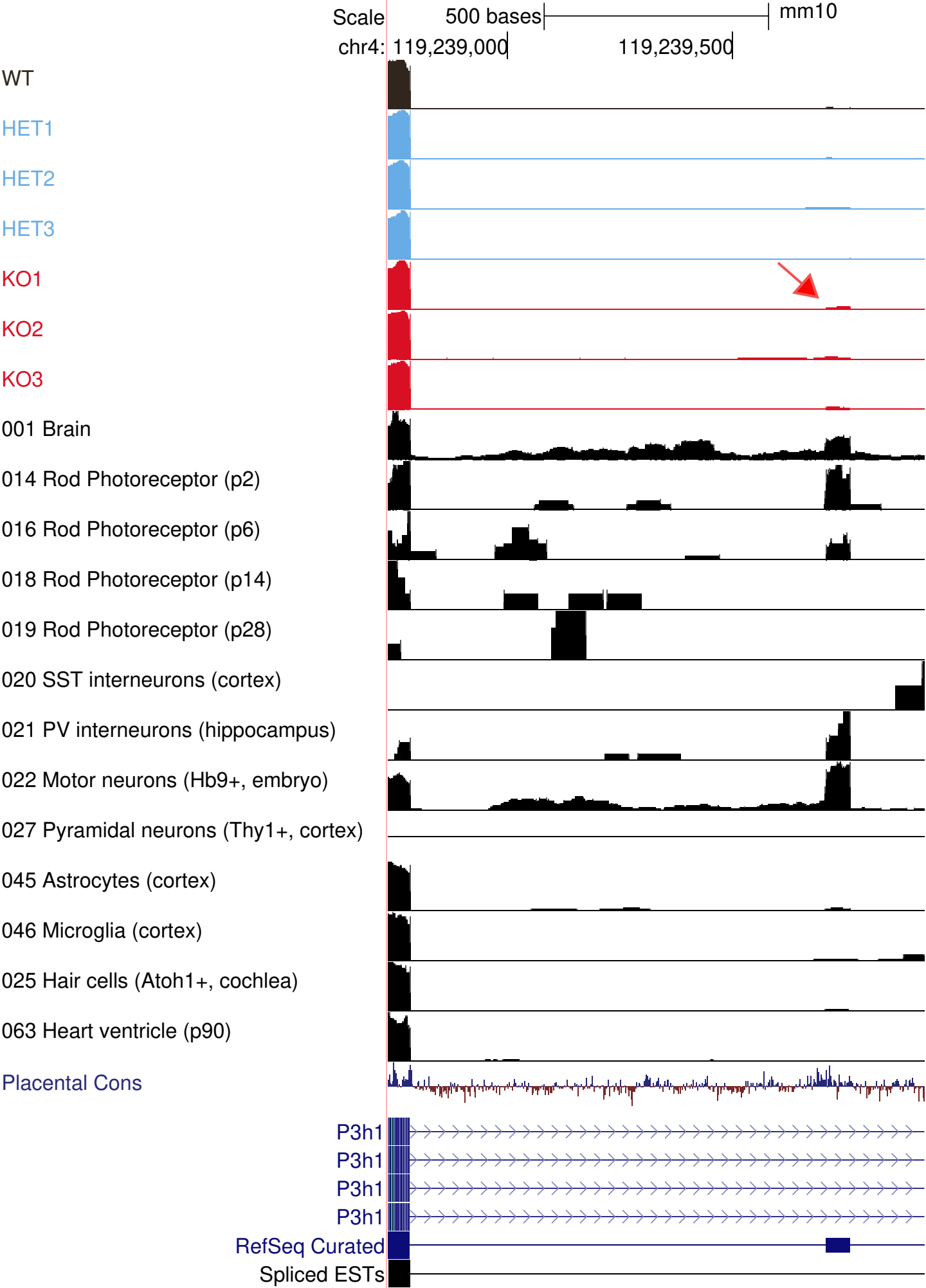

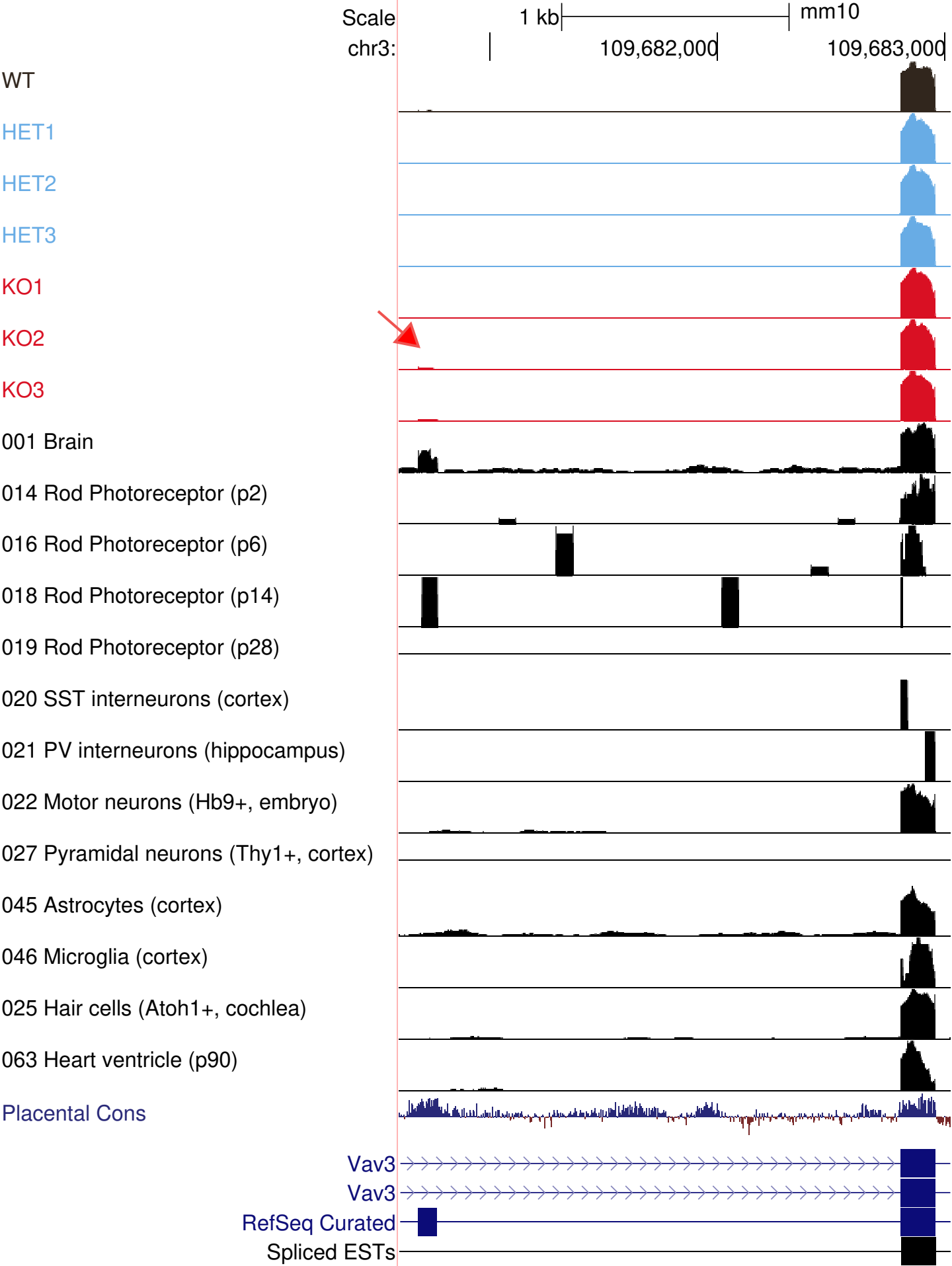

Supplement: Supplementary file 2. — Genome-browser view showing exon-level read coverage for 18 Rod-specific (A) and eight Neuron-specific (B) exons across WT, heterozygous Chx10-Cre;Ptbp1lox/l+, and homozygous Chx10-Cre;Ptbp1lox/lox mutant retinas and additional tissue, including brain, rod photoreceptors (P2, P6, P14, P28), and various neuronal and glial cell types. Tracks indicate RNA-seq read density aligned to the mm10 genome, highlighting exon-specific differences in expression and potential alternative splicing events across genotypes. [file elife-108331-supp2.pdf]
